# A variant source of arterial supply to the ascending, transverse and descending colon

**DOI:** 10.1007/s00276-019-02245-4

**Published:** 2019-04-29

**Authors:** Mange Manyama, Avelin Malyango, Ameed Raoof, Nurru L. Mligiliche, Charles Msuya, Nasnass Nassir, Estomih Mtui

**Affiliations:** 1 Medical Education Division, Weill Cornell Medicine-Qatar, Qatar Foundation, P.O. Box 24144, Doha, Qatar; 2 Department of Anatomy, Kilimanjaro Christian Medical University College, P.O. Box 2240, Moshi, Tanzania; 3 Radiology Program in Anatomy, Weill Cornell Medicine, New York, NY 10065 USA

**Keywords:** Colon, Anatomic variation, Middle mesenteric artery, Inferior mesenteric artery

## Abstract

Anatomic variations involving arterial supply of the large intestines are of clinical significance. Variations range from the pattern of origin, branching and territorial supply. The colon, the part of the large intestine, usually receives its arterial blood supply from branches of the superior and inferior mesenteric arteries. However, anatomic variation in this vascular arrangement has been reported, with vascular anatomy of the right colon being described as complex and more variable compared with the left colon. During routine cadaveric dissection of the supracolic and infracolic viscera, we encountered an additional mesenteric artery originating directly from the anterior surface of the abdominal aorta between the origins of the superior and inferior mesenteric arteries. This additional “inferior mesenteric artery” ran obliquely superiorly toward the left colon giving rise to two branches supplying the distal part of the ascending colon, the transverse colon and the proximal part of the descending colon. Awareness and knowledge of this anatomic variation are important for radiologists and surgeons to improve the quality of surgery and avoid both intra- and postoperative complications during surgical procedures of the colon.

## Introduction

The abdominal aorta usually gives rise to three anterior unpaired branches, the celiac artery (CA), superior mesenteric artery (SMA) and inferior mesenteric artery (IMA). The SMA through the ileocolic, right colic and middle colic arteries supplies structures derived from the midgut. These structures include the cecum, ascending colon and proximal two-thirds of the transverse colon. The IMA through its branches, the left colic, sigmoid and superior rectal arteries supplies structures derived from the hindgut, including the distal third of the transverse colon, descending and sigmoid colon and rectum. Anatomical variation in this classical arrangement has been reported [4, 7]. Variations in the pattern of origin, branching and territorial supply involving the CA and SMA are more common than those of the IMA [4]. Among the branches of the SMA, the ileocolic artery has been reported to be the most consistent branch and the middle colic artery the most variant branch [4]. The ascension of the left colic artery, a branch from IMA, to the left colic flexure has been reported to be limited in some cases, threatening the anastomosis of the marginal artery in this region [4].

Awareness of these and other vascular variations is of clinical significance during various surgeries involving the colon to avoid complications such as intraoperative hemorrhage and colonic ischemia [9].

The variant artery described in the present case report arose from the ventral aspect of the aorta, between the SMA and IMA and gave rise to several branches that supplied the distal part of the ascending colon, the transverse colon and the proximal part of the descending colon. Normally, these colonic regions are supplied by the middle and left colic arteries from the SMA and IMA, respectively. We discuss the possible embryological basis and the surgical implications of this vascular variation.

## Case report

In this case report, we describe a variant mesenteric artery originating directly from the abdominal aorta between the origin of the SMA and IMA in a 85-year-old Caucasian female body donor during routine dissection of the supracolic and infracolic regions. The cause of death in the donor was indicated as myocardial infarction. No past medical records were available for review.

**Fig. 1:**
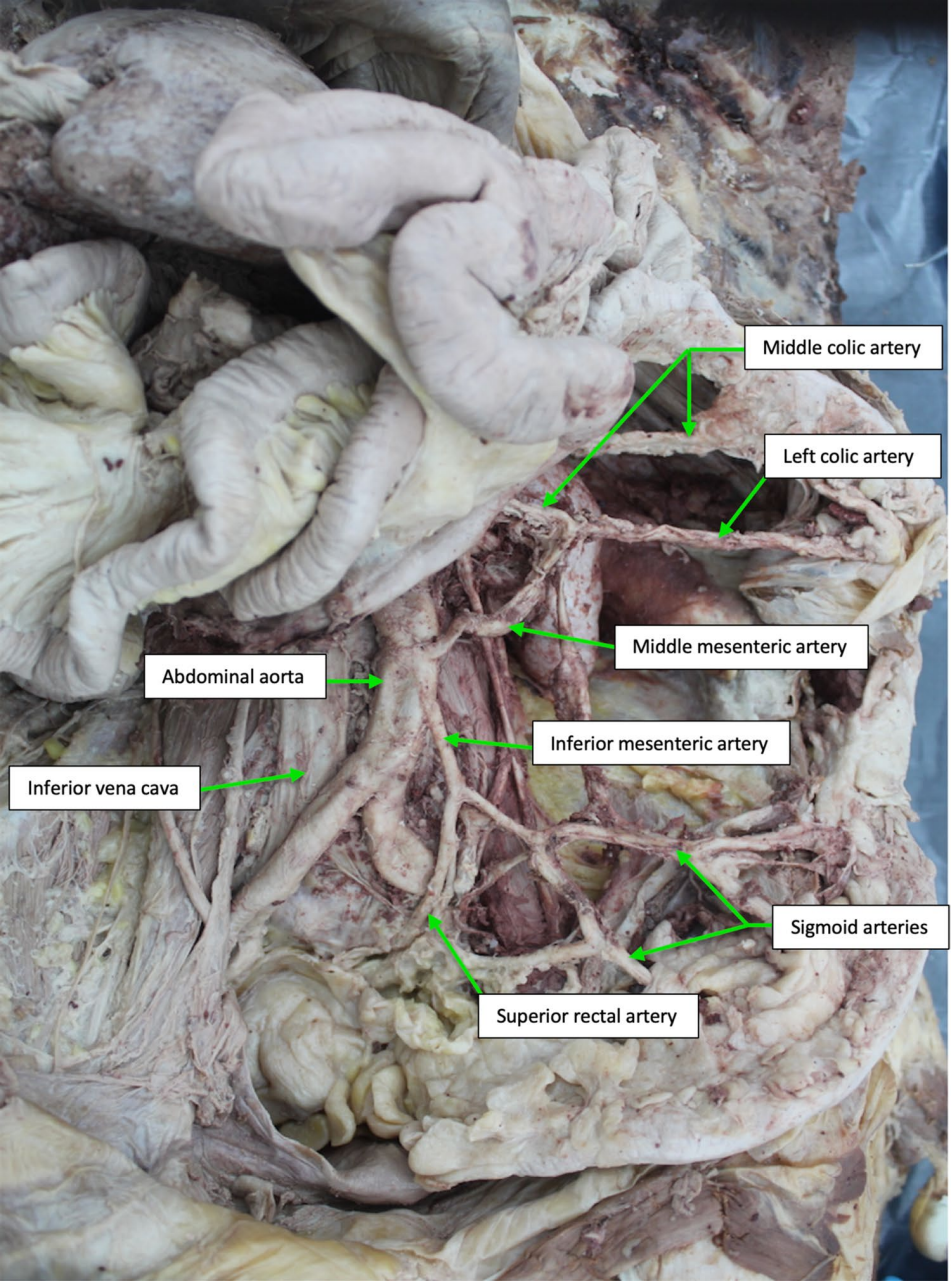
Cadaveric specimen with variant middle mesenteric artery originating directly from the anterior surface of the abdominal aorta between the origins of the superior and inferior mesenteric arteries

During the exposure of the celiac artery, superior and inferior mesenteric arteries, the intestinal loops were moved to the right and the peritoneum covering the posterior abdominal wall removed. The celiac artery and the superior mesenteric artery originated independently from the ventral surface of the abdominal aorta about 1 cm apart (Fig. 1). The CA had a normal branching pattern (Fig. 1). Branches from the SMA included the inferior pancreaticoduodenal, right colic, ileocolic, jejunal and ileal arteries. A middle colic artery that usually arises from the SMA was missing. The IMA was found to originate from the ventrolateral surface of the distal abdominal aorta about 6 cm from the origin of the SMA, and it gave rise to the sigmoid arteries and superior rectal artery. The left colic artery that usually takes its origin from the IMA was also missing.

**Fig. 2:**
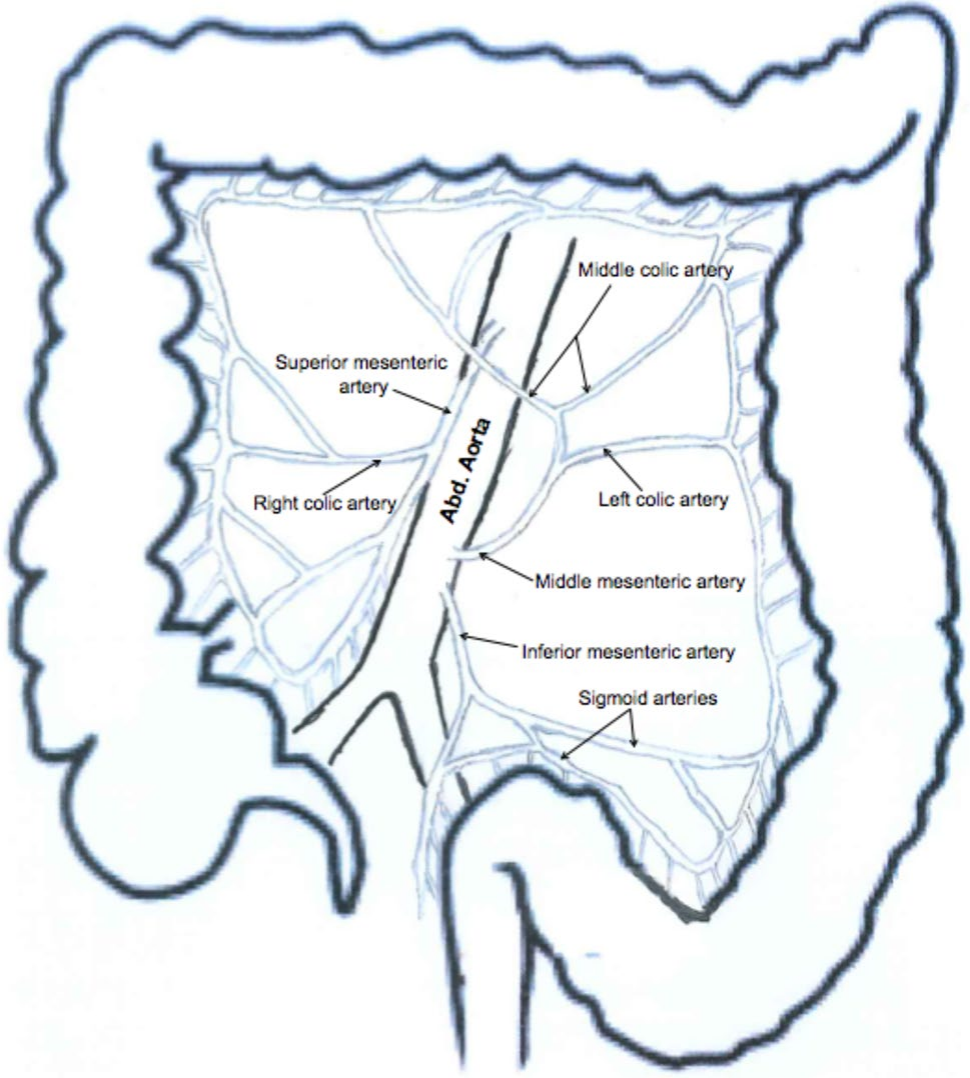
Schematic drawing of the case showing variant middle mesenteric artery originating directly from the anterior surface of the abdominal aorta between the origins of the superior and inferior mesenteric arteries. Branches of the variant artery are also shown

Between the SMA and IMA, an additional mesenteric artery was found to originate from the ventrolateral surface of the distal aorta, about 2 cm proximal to the origin of the IMA (Fig. 2). From its origin on the abdominal aorta, the variant artery ran obliquely superiorly toward the left colic flexure. It then gave rise to two branches, which had free anastomosis within the mesocolon of the transverse and descending colon. The first branch ran transversally to the left to supply the proximal part of the descending colon. The second branch ascended upward between the layers of the transverse mesocolon and gave rise to two branches that supplied the distal part of the ascending colon and the transverse colon. The distribution of the second branch is similar to the distribution of the middle colic artery, normally a branch of the superior mesenteric artery. In this case, the marginal artery of Drummond, an anastomotic vessel running in the mesentery along the inner margin of the colon, was contributed by branches from the SMA, IMA and the variant mesenteric artery.

## Discussion

The finding in this case reports a variation in the branching pattern of the arteries supplying parts of the colon. The branches supplying the distal part of the ascending colon, the transverse colon and the descending colon were found to originate from a variant branch of the abdominal aorta. Usually, the middle and left colic arteries, branches of the superior and inferior mesenteric arteries, respectively, supply these parts of the colon. The variant artery reported in this case took its origin from the abdominal aorta between the SMA and IMA before giving rise to two branches that correspond to the middle and left colic arteries. Case reports of variant arteries arising directly from the aorta between the SMA and IMA have previously been documented [5, 8].

The variations in the vascular supply of the small and large intestines can best be understood by appreciating the early development of the small and large intestines and their vascular supply. During the embryological period, each of the paired dorsal aortae gives rise to three sets of paired arterial branches: the dorsal, lateral and ventral intersegmental vessels [3]. With subsequent fusion of the dorsal aortae, several ventral branches regress and disappear and only the 10th, 13th and 21st paired ventral vessels fuse in the midline to form the celiac artery, and superior and inferior mesenteric arteries, respectively [3]. The celiac artery supplies structures derived from the foregut, while the superior and inferior mesenteric arteries supply derivatives of the midgut and hindgut, respectively. The presence of an additional third mesenteric artery in the present case arising from the aorta supplying the derivatives of the mid- and hindgut could be due to the persistence of an extra ventral intersegmental artery.

There is great similarity in the origin, course and the branching pattern of the third mesenteric artery observed in our case report and a similar case reported by Benton and Cotter [2]. Other cases with a third mesenteric artery, but with branching pattern different to our case report have also been reported [5, 8]. In these cases, the variant artery arose from the aorta between the SMA and IMA, ascended to the right and branched into the ileocolic, right and middle colic arteries. The term “middle mesenteric artery” has previously been suggested in such cases where an additional artery is found to arise from the aorta between the SMA and IMA [5, 8]. The variant artery in our case is an additional “inferior mesenteric artery”, because most of its branches supply the left colon.

The pattern of blood supply to the colon has implications on the surgical techniques and treatment outcomes on various pathological conditions involving the colon. In the surgical treatment of invasive colonic malignancies, for example, resection of the involved colon is usually accompanied with ligature of the accompanying arterial branches [1, 6]. Accidental colonic hemorrhage and ligature of anomalous arteries with subsequent infarction of the affected organs are some of the complications that could result from undetected variations in the blood supply of the colon [6]. Most of the lymphatic vessels draining the colon run in association with blood vessels, it is therefore critical for surgeons to appreciate the variations in the blood supply of the colon for maximum possible lymphatic field excision in colonic resections due to cancer.

An in-depth knowledge of the vascular anatomy of the colon and the associated pattern of collateral variation is necessary for surgeons to avoid both intra- and postoperative complications in surgical procedures involving the colon. Making sure that patients undergo radiological investigations such as selective angiography, CT and MDCT angiography that provide better visualization of vascular variations can avert some of these complications.

## References

[1] Beck DE (2002). Surgical management of colon and rectal cancer. Ochsner J.

[2] Benton RS, Cotter WB (1963). A hitherto undocumented variation of the inferior mesenteric artery in man. Anat Rec.

[3] Carlson BM (2009). Human embryology and developmental biology.

[4] De Martino RR, Oderich G (2015). Normal and variant mesenteric anatomy. Mesenteric vascular disease.

[5] Koizumi J, d’Othee BJ, Otal P, Otal P, Rousseau H, Joffre F, Kohda E, Hiramatsu K (1999). Middle mesenteric artery visualized by computed tomographic angiography. Abdom Imaging.

[6] LeQuire MH, Sorge DG, Brantley SD (1991). The middle mesenteric artery: an unusual source for colonic hemorrhage. J Vasc Interv Radiol.

[7] Michels NA, Siddharth P, Kornblith PL, Parke WW (1965). The variant blood supply to the descending colon, rectosigmoid and rectum based on 400 dissections. Its importance in regional resections: a review of medical literature. Dis Colon Rectum.

[8] Milnerowicz S, Milnerowicz A, Tabola R (2012). A middle mesenteric artery. Surg Radiol Anat.

[9] Zhang L, Ma J, Zang L, Dong F, Lu A, Feng B, He Z, Hong H, Zheng M (2016). Prevention and management of hemorrhage during a laparoscopic colorectal surgery. Ann Laparosc Endosc Surg.

